# Curvature of dendritic nonlinearities modulates higher-order spiking correlations

**DOI:** 10.1186/1471-2202-16-S1-P227

**Published:** 2015-12-18

**Authors:** Alex Cayco-Gajic, Joel Zylberberg, Eric Shea-Brown

**Affiliations:** 1 Department of Neuroscience, Physiology & Pharmacology, University College London, London, UK; 2 Department of Applied Mathematics, University of Washington, Seattle, WA, USA

## 

**Figure 1:**
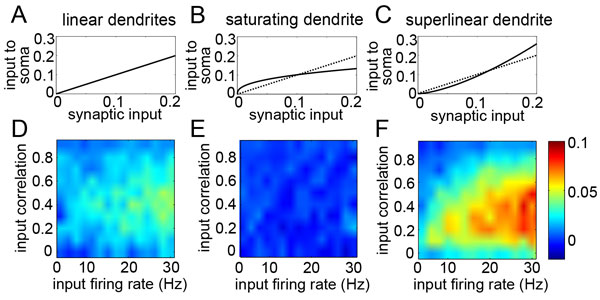
**A. Transfer functions depicting the summation of synaptic inputs for linear dendrites**. **B**. Transfer function for saturating dendritic nonlinearities (solid line), as well as the identity (dotted line). **C**. Same as **B **for superlinear dendrites. **D-F**. "Triplet correlations" (see text) between the postsynaptic cell and its presynaptic inputs, shown for **D**. linear, **E**. sublinear, and **F**. superlinear dendrites. Axes represent the varying statistics of the presynaptic spike trains, i.e., their firing rates and the correlations between them.

Characterizing neural spiking covariability is essential for understanding the collective activity of neural populations. Recent experiments have provided evidence of statistical dependencies among groups of neurons beyond that expected by the firing rates and pairwise correlations alone [1-3]. These "higher-order correlations" (HOCs) can be generated by common input [4] or motifs within the network architecture [5], yet a complete mechanistic understanding is lacking. We explore a novel mechanism through which higher-order correlations can be modulated: dendritic nonlinearities. We simulated the spiking activity of a simple exponential integrate-and-fire model neuron in response to two correlated presynaptic spike trains and background noise. The synaptic conductances were either summed linearly at the soma or were filtered through a nonlinear dendritic transfer function (see Figure 1), which was chosen to have a similar magnitude of effect on nonlinear EPSP summation as observed by active dendritic properties in pyramidal cells [6]. Using maximum entropy techniques, "triplet correlations" in the circuit were quantified as the probability of synchronous triplet spiking between the postsynaptic cell and its two presynaptic inputs beyond what could be captured by the firing rates and pairwise correlations alone, normalized by the product of the standard deviations. We found that superlinear dendrites significantly increased the level of triplet. On the other hand, saturating dendrites decorrelated triplets. These results reveal that HOCs in spiking activity are modulated by the curvature of the dendritic transfer function. Finally, this study demonstrates how intrinsic single-cell properties can tune spiking covariability in neural populations.
